# Sustainable Synthesis of Sulfur-Single Walled Carbon Nanohorns Composite for Long Cycle Life Lithium-Sulfur Battery

**DOI:** 10.3390/nano12223933

**Published:** 2022-11-08

**Authors:** Eleonora Venezia, Pejman Salimi, Susana Chauque, Remo Proietti Zaccaria

**Affiliations:** 1 Istituto Italiano di Tecnologia, Via Morego 30, 16163 Genova, Italy; 2 Department of Chemistry and Industrial Chemistry, University of Genova, Via Dodecaneso 31, 16146 Genova, Italy; 3 Department of Physics, Shaoxing University, Shaoxing 312000, China

**Keywords:** lithium–sulfur batteries, single-walled carbon nanohorns, long cycle life, sustainable synthesis process

## Abstract

Lithium–sulfur batteries are considered one of the most appealing technologies for next-generation energy-storage devices. However, the main issues impeding market breakthrough are the insulating property of sulfur and the lithium-polysulfide shuttle effect, which cause premature cell failure. To face this challenge, we employed an easy and sustainable evaporation method enabling the encapsulation of elemental sulfur within carbon nanohorns as hosting material. This synthesis process resulted in a morphology capable of ameliorating the shuttle effect and improving the electrode conductivity. The electrochemical characterization of the sulfur–carbon nanohorns active material revealed a remarkable cycle life of 800 cycles with a stable capacity of 520 mA h/g for the first 400 cycles at C/4, while reaching a value around 300 mAh/g at the 750th cycle. These results suggest sulfur–carbon nanohorn active material as a potential candidate for next-generation battery technology.

## 1. Introduction

Recent technological advancements in the electric-mobility and portable electronics fields and in smart energy grids have been driving the scientific community working in the energy-storage field towards the development and employment of higher energy density as well as more environmental friendly materials [1,2,3]. Indeed, the commercially available lithium-ion batteries (LIBs) are unable to meet these requirements due to their intrinsic limited energy density; thus, new kinds of battery technologies need to be developed [4,5]. As a result, the next generation of energy-storage systems, such as lithium–air, lithium–sulfur and sodium-ion batteries are currently under deep investigation [6,7]. In particular, lithium–sulfur batteries (LSBs) which employ elemental sulfur as cathode active material, have been widely investigated as a convincing alternative to LIBs [8]. Their electrochemical redox reaction involves the conversion of elemental sulfur S_8_ to lithium sulfide Li_2_S [9,10], thus resulting in a remarkable theoretical capacity of 1675 mAh/g and an energy density as high as 2600 Wh/kg [11,12]. Additional benefits of using sulfur as active material are its non-toxicity, wide availability, and low cost [13].

On the other hand, it is well-known that lithium–sulfur cells suffer for three main issues, namely the low conductivity of sulfur and lithium sulfide, the lithium polysulfide (LiPSs) dissolution within organic electrolyte with the associated shuttle effect, and the volume expansion of sulfur particles upon cycling [14,15,16,17]. In order to solve the aforementioned drawbacks various strategies have been introduced, such as tailoring the electrolyte composition, the application of metal oxide particles within the cathode, and the insertion of functional interlayers [18,19,20,21,22,23]. Among the most recent studies, the introduction of Al-doped ZnO particles within the sulfur cathode showed an improved cycle stability [24], while the use of Co-doped carbon composite demonstrated a prolonged cycle life [25]. All these approaches, although effective in enhancing the electrochemical performance of LSBs, require additional steps in the active material synthesis or in the cell assembly, thus resulting in an increase cell cost and augmented time-consuming production processes. Another well-explored technique capable of improving the LSBs performance is combing elemental sulfur with different kinds of host materials, mainly based on carbon, such as multi-walled carbon nanotubes, graphene, and mesoporous carbon [26,27,28,29,30]. These carbonaceous matrixes are capable of increasing the active material conductivity and reducing the detrimental effects related to the sulfur volumetric expansion, while confining or retaining the polysulfide species, thus leading to an improved cycling stability [31,32,33]. Furthermore, the associated synthesis of the composite active materials typically involves solvothermal or melting processes for which only one production step is needed [34,35]. Furthermore, strategies based on carbon composites have also been proposed to improve the LSB electrochemical performance. In this respect, carbon nanotube/nanofiber composites were investigated as a substrate for LSB cathodes, demonstrating a long-term cyclability in lean-electrolyte conditions [36], and a biomass-derived sulfur–carbon composite was employed as sulfur host exhibiting a high discharge capacity [37].

With the aim of reducing the production costs and the synthesis steps, in this work we investigated the use of single-walled carbon nanohorns (SWCNHs) as a possible sulfur host material through the implementation of a straightforward and sustainable evaporation method. SWCNHs are a class of material belonging to the fullerene family [38] and they are composed by graphitic tubules showing a peculiar horn shape. These tubules, upon aggregation, form different spherical structures, namely dahlia-like, seed-like, and bud-like structures [39]. They exhibit a good electrical conductivity, a large surface area, and high pore volume [40]. Moreover, SWCNHs can be mass-produced by arc-discharge and CO_2_ laser evaporation techniques [41,42]. Few papers report the use of SWCNHs in the battery field [43,44,45], while they have been widely studied for biosensing, drug-delivery applications, gas storage and catalysis [46,47,48,49]. We herein evaluated the application of SWCNHs as hosting material for sulfur in lithium–sulfur cells. In this work, we report on a simple solvent evaporation method enabling the easy penetration of elemental sulfur within the carbonaceous matrix. This specific morphology is able to increase the electrode conductivity and to mitigate the polysulfide shuttle effect. The outcome of the synthesis process was evaluated through scanning transmission electron microscopy-energy dispersive spectroscopy (STEM-EDX) and X-ray diffraction (XRD) measurements, which confirmed the presence of pristine sulfur within the carbonaceous structure. Therefore, the electrochemical characterization, including cyclic voltammetry, electrochemical impedance spectroscopy measurement, and lithium-ion diffusion coefficient determination was performed on the obtained electrodes. Moreover, the galvanostatic tests of the LSB cells proved a stable and long cycle life of 800 cycles, thus revealing the potential application of the sulfur-SWCNHs as a cathode in LSBs.

## 2. Materials and Methods

### 2.1. Electrolyte Preparation

The electrolyte solution was prepared by dissolving 1 mol/kg of bis(trifluoromethane)sulfonimide lithium salt (LiTFSI) in a 1:1 weight ratio solution of 1,2-dimethoxyethane (DME) and 1,3-dioxolane (DOL) in an argon-filled glovebox. Lithium nitrate (LiNO_3_) was used as additive in a concentration of 0.5 mol/kg. From here on, the electrolyte is named as DOLDME-LiTFSI-LiNO_3_. DOL and DME solvents were dried under molecular sieves for several days before mixing, while the salts were dried under vacuum at 100 °C for 24 h. Before cells were assembled, the as-prepared solution was stirred overnight. LiTFSI, DME, DOL, and LiNO_3_ were acquired from Sigma Aldrich (Merck Life Science S.r.l., Milano, MI, Italy).

### 2.2. Active Materials and Electrode Preparation

**Figure 1:**
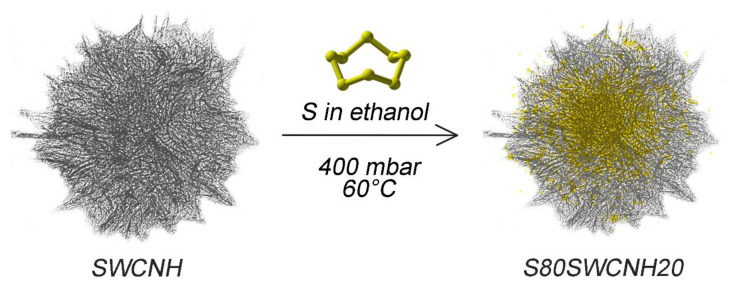
Schematic illustration of the active-material synthesis process.

The sulfur-single-wall carbon nanohorn composite (S80SWCNH20) was prepared by an easy solvent-evaporation method in order to infiltrate sulfur within the carbonaceous matrix. SWCNHs were provided by Advanced Technology Partner s.r.l.(ATP, Alessandria, AL, Italy). Elemental sulfur (from Sigma Aldrich, Merck Life Science S.r.l., Milano, MI, Italy) and SWCNHs were mixed in ethanol in a weight ratio of 80:20 and sonicated in a sonic bath for 2 h. Afterwards, the solvent was slowly evaporated at 60 °C under a pressure of 400 mbar. A schematic of the infiltration process is shown in Figure 1.

The electrode slurry was prepared by mixing S80SWCNH20 active material, Super P carbon (from Imerys, Graphite & Carbon, Willebroek, Belgium) as conductive agent, and polyvinylidene difluoride (PVdF, from Solvay, Bollate, MI, Italy) as binder in 80:10:10 weight ratio using N-methylpyrrolidone NMP (from Sigma Aldrich, Merck Life Science S.r.l., Milano, MI, Italy) as solvent. The mixture was casted onto a carbon-cloth current collector (AvCarb, from FuelCellStore, Woburn, MA, USA) by employing the doctor-blade method and dried overnight at room temperature. The electrode foil was punched into 14 mm diameter disks, dried under vacuum at room temperature overnight, and transferred in an argon-filled glovebox for cell assembly. The sulfur content within the active material was 66% while the mass loading of the final electrodes was ~2 mg/cm^2^ (total weight including the substrate ~12.5 mg).

### 2.3. Material Characterization

Thermogravimetric analysis (TGA) of the sulfur–SWCNHs composite was performed by using a Q500 thermogravimetric analyzer from TA Instruments (TA Instrument Inc., New Castle, DE, USA). The sample was heated from 30 to 600 °C at a 5 °C/min heating rate under nitrogen flow. X-ray diffraction (XRD) patterns were collected using a Malvern PANalytical Empyrean instrument (Malvern PANalytical, Malvern, United Kingdom) equipped with a Cu Kα source in the 2θ/θ scanning mode. Transmission electron microscopy (TEM), scanning transmission electron microscopy (STEM) and energy dispersive spectroscopy (STEM-EDS) images were acquired using a JOEL JEM-1400Plus (JOEL, Peabody, MA, USA) equipped with a LaB_6_ thermionic source operated at 120 kV.

### 2.4. Electrochemical Characterization

The electrochemical performance of S80SWCNH20 composite was tested in CR2032-coin cells, formed by lithium chips used as counter and reference electrode, a polymeric membrane (2400 Celgard) working as separator, and the composite electrode S80SWCNH20 as electrode. The electrolyte-to-sulfur ratio of each cell was 20 µL/mg. The assembling process was carried out in an MBraun glovebox with water and oxygen levels lower than 0.1 ppm. The electrochemical characterization was performed by using a BCS--805 multichannel battery unit from BioLogic (BioLogic, Seyssinet-Pariset, France). Galvanostatic cycling tests of the sulfur–carbon electrode were performed at the current rates of C/4 = 420 mA/g in a 1.9–2.6 V voltage range and at 1C = 1675 mA/g and 2C = 3350 mA/g in a 1.6–2.8 V voltage range. Rate capability tests were carried out at different current rates, starting from C/10 = 167.5 mA/g up to 1C = 1675 mA/g through C/8 = 210 mA/g, C/5 = 335 mA/g, C/2 = 837 mA/g, 1C = 1675 mA/g^1^, and, finally, back to C/10.

Cyclic voltammetry tests were performed at the scan rate of 0.1 mV/s over a potential rage of 1.7–2.8 V. In order to evaluate the lithium-ion diffusion coefficient (D_cv_) within the cathode material, a cyclic voltammetry test was carried out by increasing the scan rate (from 0.05 mV/s to 0.45 mV/s) in the above-reported voltage range. D_cv_ of the S80SWCNH20 cathode was calculated through the Randles–Sevcik equation [50]:

(1) $$I_{p}=0.4463\;zFAC_{Li+}\sqrt{\frac{zF\nu D_{\mathrm{CV}}}{RT}}.$$

where *I_p_* is the peak current (A), *z* is the number of electrons exchanged in the oxidation/reduction process, *F* is the Faraday constant (C/mol), *A* is the active surface area of the electrode (cm^2^), *C_Li+_* is the lithium-ion concentration in the active material (mol/cm^3^), *ν* is the voltage scan rate (V/s^1^), *R* is the universal gas constant (J/K∙mol), and *T* is the employed temperature (K), with D_cv_ calculated in cm^2^/s. 

Electrochemical impedance spectroscopy (EIS) measurements were carried out by applying a 10 mV AC amplitude signal in a frequency range of 1 MHz–0.1 Hz. The impedance spectra were fitted by Boukamp software (Equivalent Circuit 4.55, Bernard A. Boukamp, Enschede, The Netherland) [51] by non-linear leastsquares fit (NLLSQ) and only the results with a chi-square (χ^2^) lower than 10^−4^ were accepted. The equivalent circuit used to fit the data can be synthesized by the expression R_el_ (RQ)_SEI_ (R_ct_Q_dl_) Q_diff_, where R_el_ is the resistance of the electrolyte solution, (RQ)_SEI_ is attributed to the formation of the solid electrolyte interface, R_ct_ refers to the charge-transfer resistance, Q_dl_ is connected to the double-layer capacitance ascribed to the lithiation and delithiation cathode reactions, and Q_diff_ is associated with the lithium-ion diffusion into the electrode volume.

## 3. Results

### 3.1. Active Material Characterization

**Figure 2:**
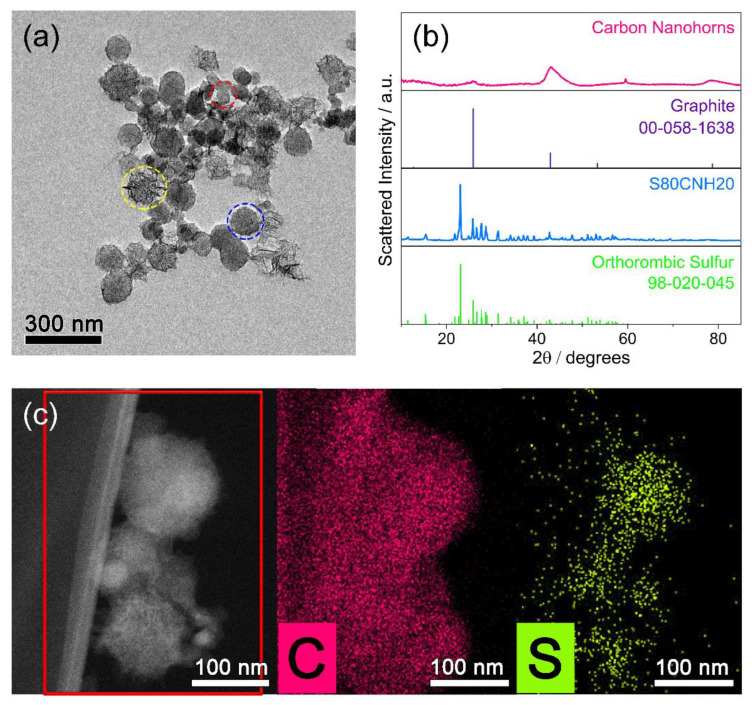
(**a**) TEM image of single-walled carbon nanohorns: dahlia-like (yellow dashed circle), bud-like (blue dashed circle), and seed-like (red dashed circle) structures. (**b**) XRD spectrum of carbon nanohorns (pink line), reference pattern of graphite (ICCD: 00-058-1638, purple bars), sulfur–carbon nanohorn composite spectrum (light blue line), and reference pattern of orthorhombic sulfur (ICDD: 98-020-045, green bars). (**c**) TEM image and STEM-EDS maps of S80SWCNH20 active material.

The S80SWCNH20 composite was prepared via a simple evaporation method and employed as the cathode active material in order to investigate its possible application in lithium–sulfur cells. The active material and the derived electrodes were analyzed by addressing their chemical structure, morphology, and electrochemical properties. The morphology of the pristine single-wall carbon nanohorns is shown in the TEM image of Figure 2a. Three different types of nanohorn aggregates were present, namely bud-like, dahlia-like, and seed-like structures, highlighted by colored dashed circles. The SWCNHs diameter ranged from 50 to 150 nm. XRD analyses were carried out in order to confirm the presence and the crystalline phase of sulfur within the synthesized active material. Figure 2b reports the XRD spectra of pure SWCNHs and of the composite material together with the reference pattern of graphite and sulfur. The XRD spectrum of the SWCNHs reveals the presence of the characteristic peaks of graphite (ICCD: 00-058-1638, pink bars) where the peaks at 23° and about 43° can be attributed to the (002) and (10) reflections [38]. The pattern of the composite active material, in light blue, shows the presence of the peaks attributed to orthorhombic sulfur (ICDD: 98-020-045, green bars) overlapped with the weaker SWCNHs broad peaks. In order to characterize the morphology of the composite sample after the synthesis and further verify the presence of sulfur within the SWCNHs, a STEM-EDS analysis was carried out and the results are reported in Figure 2c. The dark-field TEM image of the S80SWCNH20 composite shows the three CNHs species, as also confirmed by the elemental mapping of carbon (pink), and highlights the absence of isolated sulfur aggregates. In this respect, the corresponding elemental mapping of sulfur (green) evidences the presence of a high sulfur concentration inside the carbon nanohorn structure. These observations suggest the presence of a morphology capable of encapsulating the dissolved LiPS species thus ameliorating the detrimental shuttle effect [52,53].

**Figure 3:**
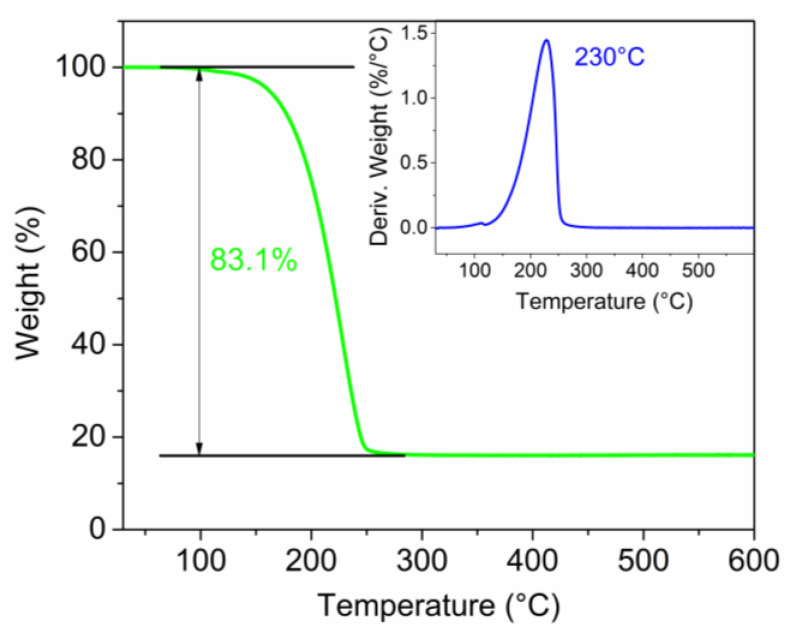
TGA of the composite active material S80SWCNH20.

The sulfur–carbon nanohorn composite was further investigated by TGA to find out its exact sulfur content. The measurement was carried out under argon in a 30–600 °C temperature range. Figure 3 shows the TGA profile, revealing an overall sulfur content of about 83% within the composite active material (66.4% of sulfur within the final electrode), while the remaining mass corresponds to the carbonaceous material. Thus, the simple evaporation method herein reported enabled the easy control of the sulfur and carbon amount. In the inset, the relative differential profile evidences a sulfur-evaporation temperature of 230 °C.

### 3.2. Electrochemical Characterization

**Figure 4:**
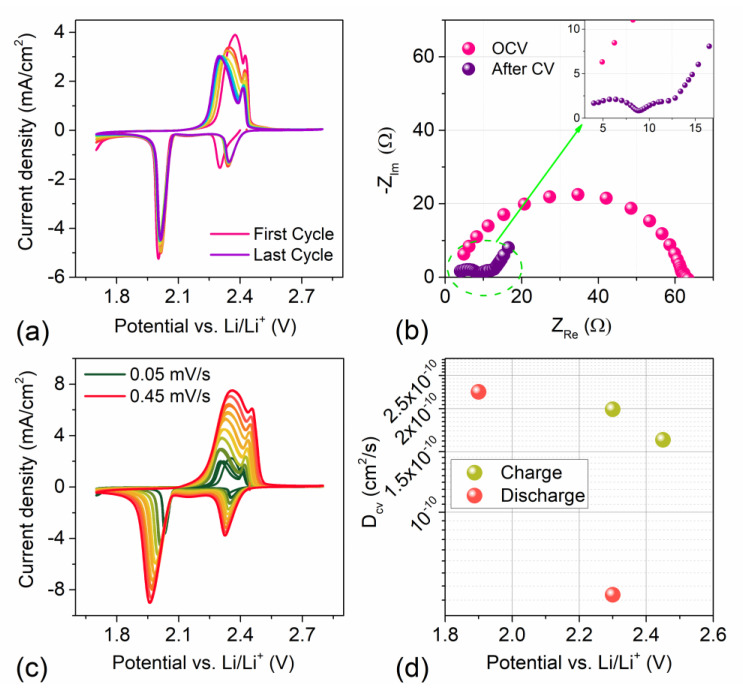
(**a**) Cyclic voltammetry (CV) test of S80CNH20 electrode, performed in 2032-coin cells using DOLDME-LITFSI-LiNO_3_ as electrolyte in 1.7 V–2.8 V voltage range with a scan rate of 0.1 mV/s. (**b**) EIS conducted as assembled and after the cyclic voltammetry. (**c**) CV tests carried out at increasing scan rate (from 0.05 to 0.45 mV/s) in order to calculate the lithium diffusion coefficient D_cv_ within the electrode material through the Randles–Sevcik equation (see Equation (1) and Figure S2 in the Supplementary Materials). (**d**) Lithium-ion diffusion coefficients obtained by using the Randles–Sevcik equation, the peak intensity (*I_p_*) and the scan rate (ν).

The electrochemical behavior of the S80SWCNH20 composite electrode was explored using 2032-coin cells. DOLDME-LITFSI-LiNO_3_ was employed as electrolytic solution in order to probe the performance of the electrode in lithium–sulfur batteries. Figure 4a reports the cyclic voltammetry curves (10 cycles) presenting the typical shape attributed to lithium–sulfur reactions within the selected electrolyte. Indeed, the cathodic scan shows a peak at 2.35 V was related to the conversion of S_8_ rings to long-chain lithium polysulfide species Li_2_S_x_ (6 < x ≤ 8) while the second sharp peak at about 1.90 V suggests the reduction of the long-chain LiPSs to short-chain ones, i.e., Li_2_S_x_ (2 < x ≤ 6) and Li_2_S [12,54]. Similarly, the anodic curve shows the first peak at ~2.35 V, which could be attributed to the oxidation of short chain LiPSs to higher-order species, while the second peak at 2.45 V was related to the formation of long-chain polysulfide and, finally, of elemental sulfur [55]. The test evidenced a decrease in the peaks’ intensities during the first five CV cycles, indicating a possible polysulfide dissolution within the electrolyte thus leading to active material loss [56]. After this initial intensity drop, the CV curves overlap one another, thus suggesting cell stabilization upon cycling. EIS measurements were carried out before and after the CV test to investigate the internal resistance changes upon cycling. The associated Nyquist plot is reported in Figure 4b. The impedance spectrum at the fresh state shows a broad and depressed semicircle with an overall resistance of 60 Ω which evolves into two semicircles after the CV test, revealing a total resistance of 15 Ω (see inset of Figure 4b). This value is indeed the combination of the electrolyte resistance (R_el_ = 3 Ω), the SEI resistance ((RQ)_SEI_ = 5.5 Ω), and the charge-transfer resistance (R_ct_ = 7.5 Ω). The first and the second semicircles can be ascribed to the formation of the solid electrolyte interphase (SEI) on the electrode surface and to the charge-transfer processes occurring at the interface between electrode and electrolyte, respectively [4]. Indeed, the decrease of the cell resistance suggests the formation of a low resistive SEI facilitating the lithium-ion transfer [57].

Figure 4c reports the CV tests performed by increasing the scan rate from 0.05 to 0.45 mV/s^1^ in order to calculate D_cv_ within the electrode material (see Figure S1 of Supplementary Materials for EIS after each incremental step). As expected, by increasing the scan rate, the peak intensity increased accordingly due to the decreased size of the diffusion layer [58]. Moreover, increasing the scan rate determines a reduction of the cathodic peak potential and an increase of the anodic peak potential, a result due to mass transfer limitation [59]. D_cv_ was obtained through the Randles–Sevcik equation described in Equation (1). This equation linearly correlates the peak current intensity (*I_p_*) to the square root of the scan rate (ν^1/2^) (see Figure S2 of Supplementary Materials), where the slope value is dependent on the lithium diffusion coefficient [60]. D_cv_ was calculated for different states of charge (2.35 and 2.45 V) and discharge (1.90 and 2.35 V) considering two electrons for each oxidation/reduction process. The results are reported in Figure 4d and, in detail, the D_cv_ added up to 2 ∙ 10^−10^ and 1.6 ∙ 10^−10^ cm^2^/s for the oxidation reaction occurring at 2.35 V and 2.45 V, respectively. Upon reduction, the lithium-ion diffusion appeared to be slower for the reduction of S_8_ to long-chain LiPSs at 2.35 V with respect to the reduction to short-chain LiPSs occurring at 1.90 V, showing D_cv_ values of about 5 ∙ 10^−11^ and 2.2 ∙ 10^−10^ cm^2^/s, respectively, in agreement with the CV test.

**Figure 5:**
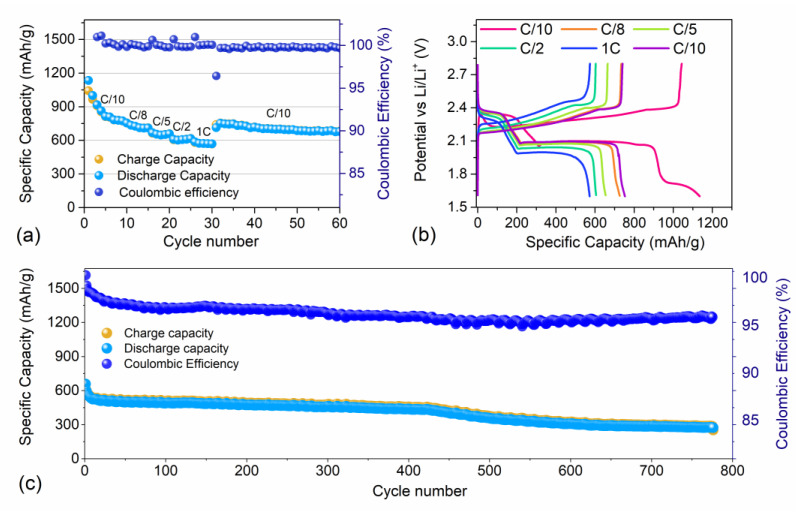
(**a**) Rate capability test and (**b**) relative voltage profiles of S80CNH20 performed at different current-rate: C/10 to 1C (=1675 mA/g) through C/8, C/5, C/2, and 1C and finally back to C/10. Sulfur loading of ~2 mg/cm^2^. (**c**) Galvanostatic cycling profile of the electrode performed in 2032-coin cells with DOLDME-LITFSI-LiNO_3_ electrolyte carried out at C/4= 419 mA/g within 1.9 V and 2.6 V. Sulfur loading of ~2 mg/cm^2^ (see Figure S4 in Supplementary Material for voltage profiles).

Rate capability and galvanostatic cycling tests were performed on the S80SWCNH20 electrode and are reported in Figure 5. In particular, Figure 5a,b display the rate capability tests performed by increasing the current rate from C/10 = 167.5 mA/g up to 1C = 1675 mA/g through C/8 = 210 mA/g, C/5 = 335 mA/g, C/2 = 837 mA/g, 1C = 1675 mA/g, and finally back to C/10 in a 1.6 V–2.8 V voltage range. The tests were carried out to understand the cell behavior at different current rates. As seen from Figure 5a, the cell specific capacity decreased rapidly at C/10 during the first five cycles from 1000 mAh/g (considering the second discharge cycle, while 1135 mAh/g was achieved at the first cycle) down to 735 mAh/g, followed by a stabilization in the capacity values. By increasing the current rate, the cell polarization between charge and discharge curves increased from 0.2 V at C/10 to 0.4 V at 1C (Figure 5b) thus leading to a decrease in the cell-specific capacity. Indeed, at 1C the delivered capacity was reduced to 570 mAh/g. Once the current was set back to its original value (C/10), the cell recovered and maintained the specific capacity reached after the initial drop (~755 mAh/g). The initial fast capacity fading, well recognizable in Figure 5a, can be ascribed to the presence of elemental sulfur on the carbon nanohorn surface, leading to the dissolution of LiPSs within the electrolyte and consequently to active material losses [61]. On the other hand, the subsequent stabilization in the specific capacity values could suggest LiPS retention inside the SWCNHs, the latter acting as a physical barriers, thus limiting the shuttle effect [62]. The Coulombic efficiency of the S80SWCNH20 cell was maintained at a stable value of 99.6% during the whole test. The voltage profiles (Figure 5b) showed the characteristic curves attributed to the electrochemical reactions between lithium and sulfur. Indeed, two plateaus at 2.4 V and at 2.1 V during the discharge process were present, indicating the reduction of elemental sulfur to Li_2_S discharge product. Upon charging, a long plateau was visible at 2.2 V, which increased to 2.35 V during the test, followed by a second short plateau at 2.4 V. The plots well highlight the cell polarization upon increasing the current rate. A prolonged galvanostatic cycling test was carried out at C/4 and it is reported in Figure 5c. A similar behavior to the rate capability test, with an initial capacity drop followed by a stabilization in the capacity values, was observed here. Indeed, the initial specific capacity of 600 mAh/g decreased to 520 mAh/g in the first 10 cycles and remained stable for 400 cycles. Subsequently, the cell capacity slowly reduced reaching a value of about 300 mAh/g at the 775th cycle, which could be attributed to the formation of a ‘dead sulfur’ layer and an increase in the cell polarization, as it can be seen from the voltage profiles in Figure S3 [63]. The Coulombic efficiency of the S80SWCNH20 cell revealed a gradual decrease during the prolonged cycling test. This effect could be related to a mild polysulfide shuttle effect which consumes the active material during the charge/discharge reactions [64].

These results suggest the morphology as a crucial parameter in order to stabilize the cell performance. Galvanostatic charge and discharge measurements were carried out on the S80SWCNH20 electrodes at the high current rate of 1C (1.675 A/g) and the results are reported in Figure S4 of Supplementary Materials. The test shows a high specific capacity if considering the high current rate, which could be ascribed to the high electronic conductivity of the carbon nanohorns combined with an optimized active material morphology.

Overall, the S80SWCNH20 cells demonstrated a long cycle life without the addition of catalysts, functional materials, or the use of a complicated synthesis. The simple, easy, and sustainable synthesis process herein employed to prepare the cell active material proved to be an effective way to produce a long-cycle-life sulfur-based electrode.

Finally, Table 1 reports a comparison of the cycling performance of recently proposed sulfur composites. The data suggest the active material introduced in this work to be a promising and sustainable solution to achieve long-cycle-life LSBs with remarkable specific capacity values.

## 4. Conclusions

We have herein demonstrated an easy synthesis process to produce a sulfur–carbon nanohorn active material for next-generation lithium–sulfur batteries. The straightforward and sustainable evaporation method reported in this work allows for the direct infiltration of elemental sulfur within single-walled carbon nanohorns. Moreover, the same synthesis process enables easy control of the sulfur and carbon amount within the active material. In this respect, the sulfur-based electrodes were electrochemically tested in lithium-metal half-cells showing a long and stable cycle life. This interesting performance can be attributed to the material morphology, which enables the important polysulfide retention by physically confining the soluble moieties within the SWCNH structures, the latter also improving the electrode conductivity. The straightforward synthesis of the sulfur carbon nanohorn composite makes this material a feasible alternative for the cathode in lithium–sulfur batteries.

## Figures and Tables

**Table 1:** Comparison of the main electrochemical criteria of some recently published papers regarding sulfur composite cathode in lithium–sulfur battery.

Cathode	Initial Discharge Capacity (mAh/g^1^) at Low C-Rate	Cycle Life (Cycles)	C-Rate	Specific Discharge Capacity after Cycling (mAh/g)	Reference
S80SWCNH20	1135	775	C/4	300	**This work**
Ti_3_C_2_Tx@S	~1250	200	C/2	429	[65]
MPC–6S	931	400	C/2	~200	[66]
Biomass-derived carbon/sulfur	1067	500	C/10	254	[37]
3DGNs/S	790	280	1C	~400	[67]
S/MPC	~1600	70	0.2 A·g^−1^	~500	[68]
Sulfur/MBGO20	~900	100	C/5	~400	[69]
Ta_2_O_5_@C/S	~1250	300	C/2	~400	[70]
S/ZIF-67	968	1000	1C	237	[25]
S-Nb_4_N_5_	~1200	400	1C	~300	[71]
MIL-88A@S	600	1000	C/2	~300	[72]
S/RGO@NPC	~1200	400	C/2	600	[73]
CoP-CNT@C/S	1457	750	C/2	474	[74]
S@d-MXene	769	500	1C	506	[75]
MMC@S	1124	60	C/5	616	[76]
NT/MnO_2_-S	704	300	C/2	429	[77]

## Data Availability

Not applicable.

## Supplementary Materials

The supporting information can be downloaded at: https://www.mdpi.com/article/10.3390/nano12223933/s1, Figure S1: EIS of the S80SWCNH20 electrode acquired at each scan rate, from 0.05 to 0.45 mV/s during the cyclic voltammetry tests; Figure S2: Linear fit of the peak current (*I_p_*) plotted vs. square root of the scan rate (ν^1/2^) of the cyclic voltammetry of Figure 4c of the S80SWCNH20 electrode for different states of charge; Figure S3: Voltage profiles of S80SWCNH20 performed at C/4 (=419 mA/g) in a 1.7–2.8 V voltage range; Figure S4: Galvanostatic cycling test of the S80SWNH20 electrode carried out at 1C (=1675 mA/g).
