# Supplementary material for: Sustainable Synthesis of Sulfur-Single Walled Carbon Nanohorns Composite for Long Cycle Life Lithium-Sulfur Battery
Source: Nanomaterials (Basel). 2022 Nov 8;12(22):3933. doi: 10.3390/nano12223933 (PMC9699005; doi:10.3390/nano12223933)
Supplement: Supplementary file 1 [file nanomaterials-12-03933-s001.zip › nanomaterials-2010622-supplementary.pdf]

Supporting Materials

Sustainable Synthesis of Sulfur–Single Walled Carbon Nanohorns Composite for Long Cycle Life Lithium–Sulfur Battery

Eleonora Venezia [1,2], Pejman Salimi [1,2], Susana Chauque [1], Remo Proietti Zaccaria [1,3,*]

[1] Istituto Italiano di Tecnologia, via Morego 30, Genova 16163, Italy
[2] Department of Chemistry and Industrial Chemistry, University of Genova, via Dodecaneso 31, I-16146 Genova, Italy
[3] Department of Physics, Shaoxing University, Shaoxing 312000, China
* Correspondence: remo.proietti@iit.it; Tel.: +39 331 2576045

Figure S1 shows the electrochemical impedance spectroscopy measurements obtained after each incremental scan step during the cyclic voltammetry (CV) test reported in the Manuscript as Figure 2(c). The appearance of two depressed semicircles after the first CV cycle suggests the formation of an SEI on the electrodes surface thus improving the lithium ion charge transfer.

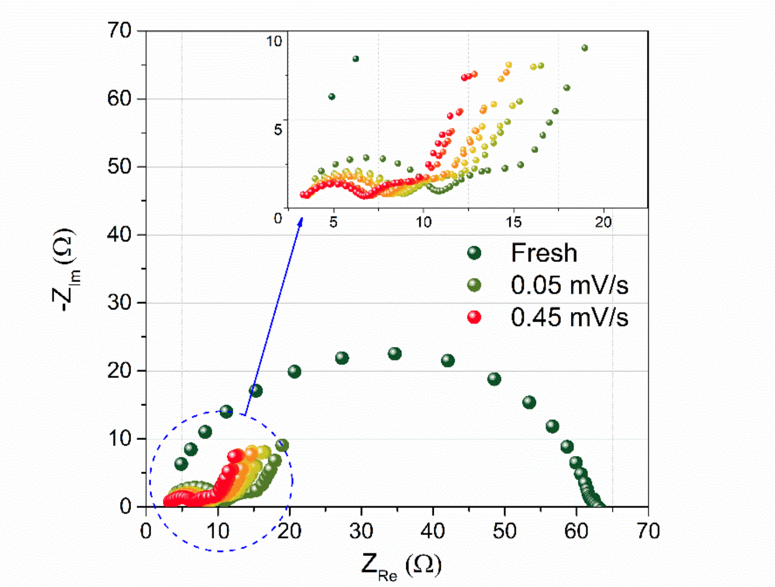

Figure S1. Electrochemical impedance spectroscopy (EIS) spectra of the S80SWCNH20 electrode acquired at each scan rate, from 0.05 to 0.45 mV/s along the cyclic voltammetry tests of Figure 2(c).

Figure S2 was obtained from the cyclic voltammetry profiles of Figure 2(c) and represent the linear fit of charge and discharge peak current (I_P) vs. the square root of the scan rate ($\nu^{1/2}$). The Figure reports the fits of charge (2.3 and 2.4 V vs. Li^+/Li) and discharge peaks (1.3 and 1.9 V vs. Li^+/Li) considering 2 electrons for

each peak. The slope of the linear fits was used to calculate the lithium diffusion coefficient (see Figure 2(d) of the Manuscript) according to the Randles-Sevcik equation (see Manuscript Eq. 1).

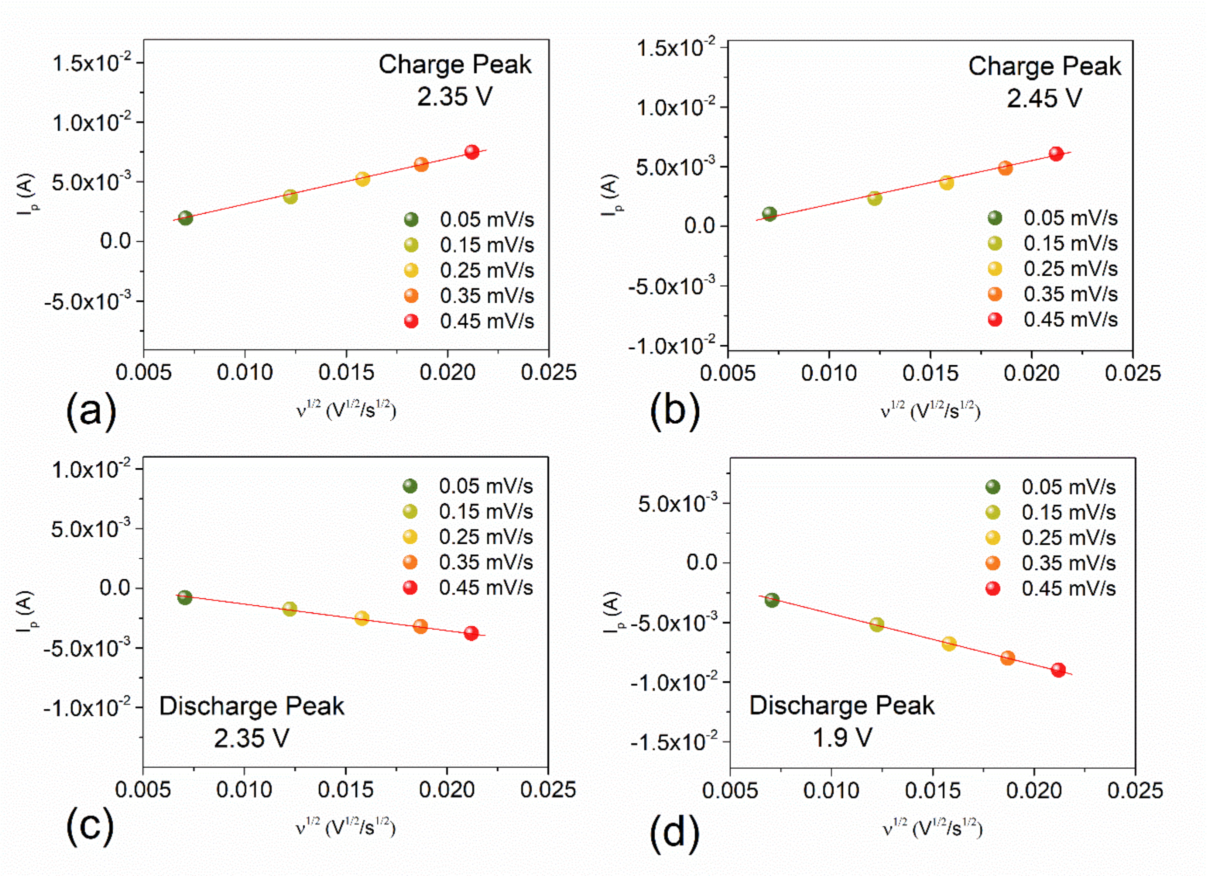

Figure S2. Linear fit of the peak current (I_p) plotted vs. square root of the scan rate ($\nu^{1/2}$) of the cyclic voltammetry of Figure 4(c) of the S80SWCNH20 electrode for different states of charge.

The voltage profiles relative to the galvanostatic test at C/4 of the S80SWCNH20 electrode are reported in Figure S3. The cell demonstrated a stable capacity over 400 cycles, which then decreases reaching a value of about 300 mA h/g at the 775th cycle. During the test, the cell polarization increases, thus causing a drop in the specific capacity value.

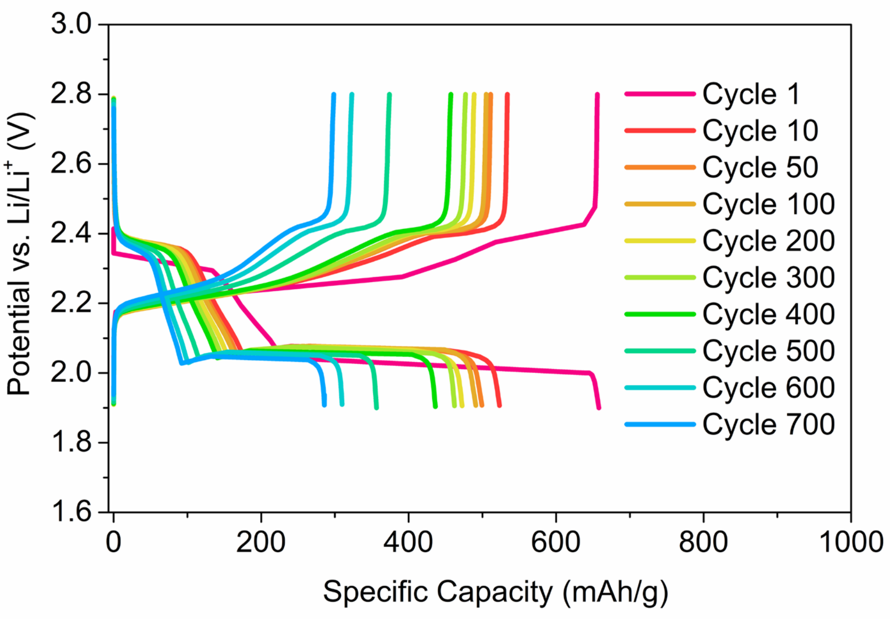

Figure S3. Voltage profiles of S80SWCNH20 performed at C/4 (= 419 mA/g) in a 1.7–2.8 V voltage range relative to Figure 3(c) of the Manuscript.

The S80SWCNH20 electrode was tested at 1C to evaluate its performance at high current rate and the results are reported in Figure S4. The test presents a similar profile involving a slow capacity drop along the charge and discharge cycles. At 1C, the cell presents an initial capacity of 770 mA h/g which then reaches to about 500 mA h/g after 160 cycles.

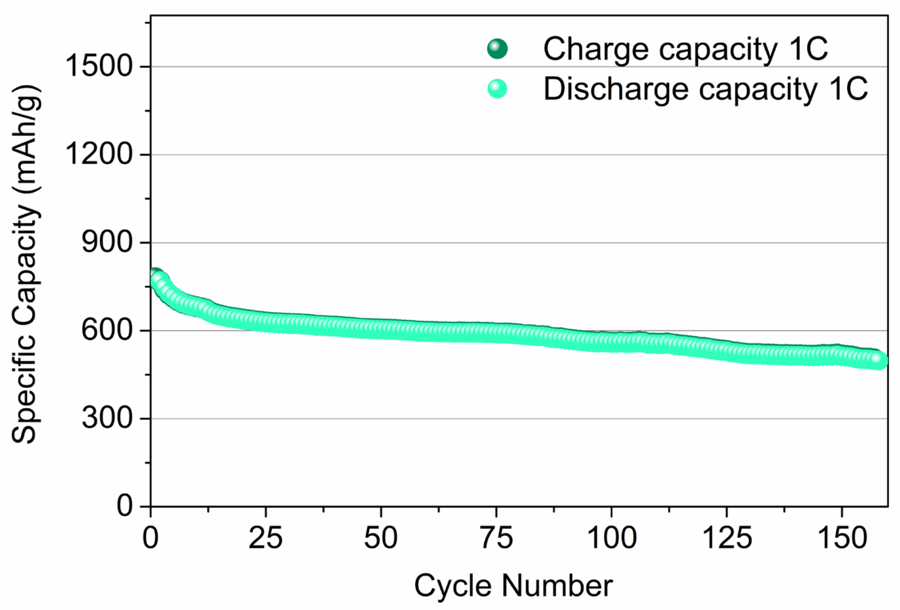

Figure S4. Galvanostatic cycling test of the S80SWNH20 electrode carried out at 1C (= 1675 mA/g) within a 1.6−2.8 V voltage range. Sulfur loading = 1.6 mg/cm^2.
